# Adherent Human Alveolar Macrophages Exhibit a Transient Pro-Inflammatory Profile That Confounds Responses to Innate Immune Stimulation

**DOI:** 10.1371/journal.pone.0040348

**Published:** 2012-06-29

**Authors:** Gillian S. Tomlinson, Helen Booth, Sarah J. Petit, Elspeth Potton, Greg J. Towers, Robert F. Miller, Benjamin M. Chain, Mahdad Noursadeghi

**Affiliations:** 1 Infection and Immunity, University College London, London, United Kingdom; 2 Centre for Respiratory Research, University College London, London, United Kingdom; 3 Department of Thoracic Medicine University College London Hospitals NHS Trust, London, United Kingdom; 4 Research Department of Infection and Population Health, University College London, London, United Kingdom; Centre of Influenza Research, The University of Hong Kong, Hong Kong

## Abstract

Alveolar macrophages (AM) are thought to have a key role in the immunopathogenesis of respiratory diseases. We sought to test the hypothesis that human AM exhibit an anti-inflammatory bias by making genome-wide comparisons with monocyte derived macrophages (MDM). Adherent AM obtained by bronchoalveolar lavage of patients under investigation for haemoptysis, but found to have no respiratory pathology, were compared to MDM from healthy volunteers by whole genome transcriptional profiling before and after innate immune stimulation. We found that freshly isolated AM exhibited a marked pro-inflammatory transcriptional signature. High levels of basal pro-inflammatory gene expression gave the impression of attenuated responses to lipopolysaccharide (LPS) and the RNA analogue, poly IC, but in rested cells pro-inflammatory gene expression declined and transcriptional responsiveness to these stimuli was restored. In comparison to MDM, both freshly isolated and rested AM showed upregulation of MHC class II molecules. In most experimental paradigms *ex vivo* adherent AM are used immediately after isolation. Therefore, the confounding effects of their pro-inflammatory profile at baseline need careful consideration. Moreover, despite the prevailing view that AM have an anti-inflammatory bias, our data clearly show that they can adopt a striking pro-inflammatory phenotype, and may have greater capacity for presentation of exogenous antigens than MDM.

## Introduction

Tissue resident macrophages play a key role in host defense by generating potent inflammatory responses to innate immune stimulation, but also regulate anti-inflammatory mechanisms which are crucial for resolution of inflammatory responses and restoration of tissue homeostasis. Alveolar macrophages (AM) are of specific interest as the principal cellular sentinels of the distal respiratory tract, because they are continuously exposed to potentially inflammatory stimuli, within a delicate tissue microenvironment that is particularly susceptible to damage. Innate immune responses by AM are crucial for immunosurveillance and host protection against inhaled pathogens, but excessive inflammatory responses by these cells also underpins the pathogenesis of numerous diseases including sarcoidosis, pulmonary fibrosis, tuberculosis and *Streptococcus pneumoniae* infection [1]–[4].

Inflammatory responses by macrophages are subject to extensive research, but findings are commonly extrapolated from experiments with peripheral blood monocyte derived macrophages (MDM). In humans, AM can readily be obtained by bronchoalveolar lavage (BAL), providing a population of genuine primary tissue macrophages which are amenable to study. Experimental observations are often interchangeably extrapolated between these two common cellular models, but in view of the inherent phenotypic heterogeneity between different macrophage populations the extent to which this is appropriate is uncertain. By comparison with other macrophages, AM are reported to exhibit a highly specialised phenotype characterised by enhanced pattern recognition receptor (PRR) and scavenger receptor expression combined with poor antigen presentation capacity [5]–[7], which is specifically adapted for clearance of inhaled particulates and controlled orchestration of inflammatory responses via recruitment of professional antigen presenting cells (APC) from the circulation, whilst also limiting indiscriminate inflammation.

Macrophage development *in vivo* is thought to be driven principally by endocrine function of colony stimulating factor (CSF)1, also known as macrophage (M–CSF, and its cognate receptor- CSF1R [8], hence M–CSF is widely used to generate MDM. Previous comparisons of MDM and AM have involved only very limited assessment of cell surface markers and some functional observations. The surface antigen expression profile of AM (CD14 low, CD16 high, CD71 positive, high MHC class II) has previously been reported to closely resemble that of MDM differentiated with granulocyte macrophage (GM)-CSF [9]. Moreover, GM-CSF is essential for terminal differentiation of AM driven by PU.1- a key transcriptional regulator of macrophage differentiation [10], and deficient GM-CSF function is associated with alveolar proteinosis as result of impaired surfactant catabolism by AM [11]. Collectively these data suggest that GM-CSF is physiologically important for the homeostatic phenotype and function of AM, but in the wider context, GM-CSF is a component of the host immune response and is known to modulate MDM function towards a so-called M1 phenotype which exhibit enhanced pro-inflammatory responses, yet the prevailing view is that AM have predominantly anti-inflammatory properties on the basis of prostaglandin E2 (PGE2), TGFβ and IL10 production and their ability to suppress T cell proliferation *in vitro* [12], [13].

In view of this paradox, we sought to reassess the phenotype of AM and their responses to innate immune stimulation, in comparison to MDM, by a systems biology approach using genome-wide transcriptional profiling and testing gene expression changes invoked by the prototypic inflammatory stimulus, lipopolysaccharide (LPS).

## Results

### AM and MDM Share Common Phenotypic Characteristics

**Figure 1:**
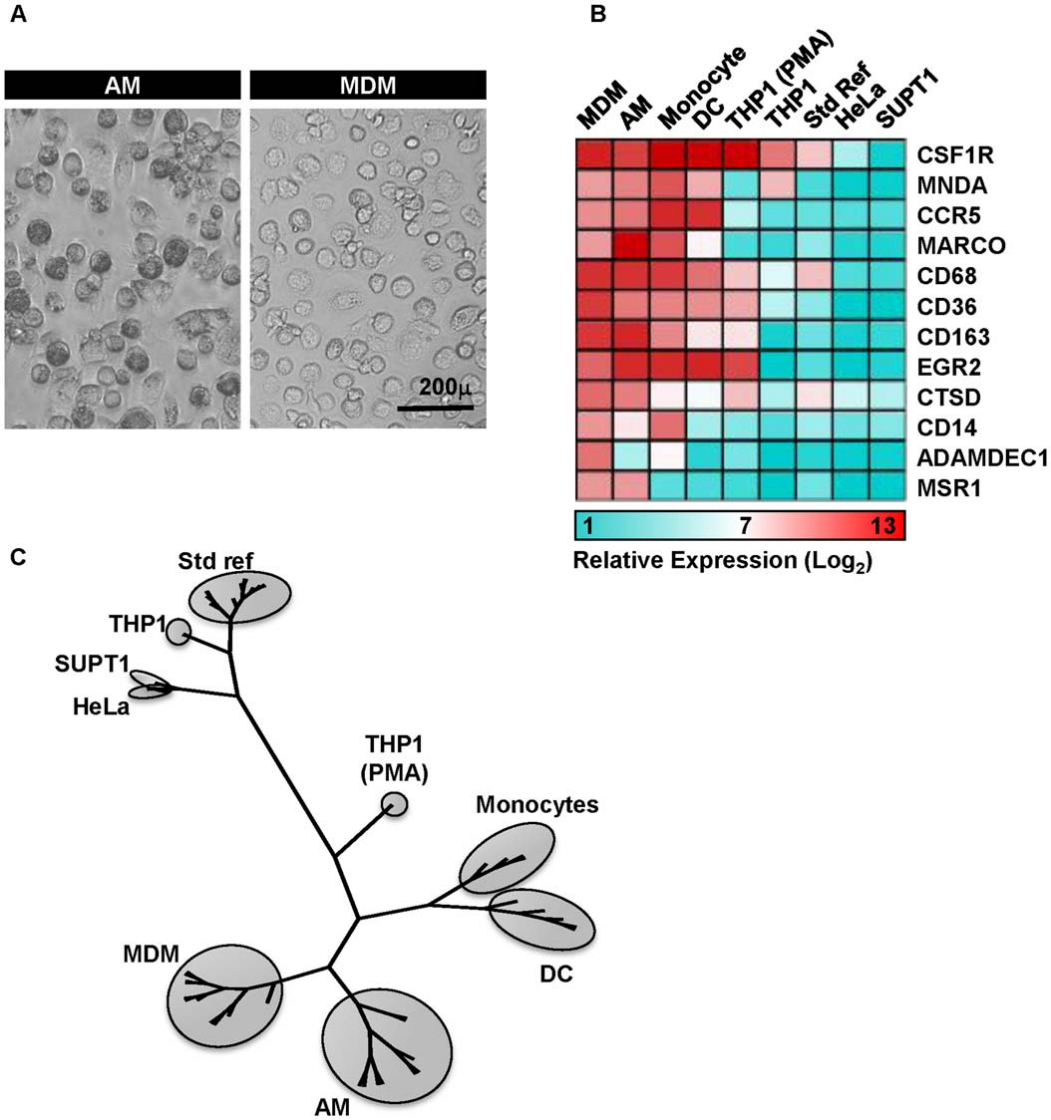
AM and MDM share common gene expression characteristics. (A) AM cultured for 24 hours exhibit similar appearances to MDM (differentiated for seven days) on light microscopy. (B) Mean relative gene expression levels of selected macrophage characteristic genes are comparable in AM (from four donors) and MDM (from eight donors). Many of these genes are also expressed in monocytes (from four donors), and to a lesser extent in DC (from five donors), but not in any of the cell lines. (C) Hierarchical clustering on the basis of macrophage signature genes also shows that AM and MDM transcriptional profiles are closely related, and segregate together with other myeloid cells, distinct from undifferentiated THP1, HeLa and SUPT1 cells and a standard reference RNA sample.

Bronchoscopy and bronchoalveolar lavage are commonly used for investigation of haemoptysis with normal radiographic imaging, in order to exclude respiratory tract pathology [14]. This provides an invaluable opportunity to obtain AM from healthy donors. Subjects included in this study were specifically selected on the basis that there was no clinical or laboratory evidence for ongoing respiratory tract pathology, and in particular that respiratory infection was excluded. Bronchoscopy was performed to exclude neoplasia as a cause of haemoptysis. Demographic and clinical data for these subjects is presented in Table S1. AM had a similar morphological appearance to MDM (Figure 1A). We next sought to confirm their relationship to the macrophage lineage at a molecular level, by identifying a transcriptional signature for macrophages from whole genome expression profiling (Table S2). Expression levels of these genes in AM were compared to MDM, monocytes, monocyte derived DC (MDDC), universal standard reference RNA and three human cell lines- THP1, derived from a myeloid leukaemia, HeLa, derived from carcinoma of the cervix and SUPT1, derived from a T cell lymphoblastic lymphoma (Figure 1B). Overall, AM, MDM, monocytes and DC all showed similar expression levels for this macrophage-associated gene signature, in stark contrast to the other cell types. Hierarchical clustering analysis of expression data for these macrophage signature genes showed AM to cluster most closely with MDM and segregated, together with monocytes, DC and phorbol myristate acetate-differentiated THP1 cells from standard reference RNA, HeLa and SUPT1 cells, and undifferentiated THP1 cells (Figure 1C).

### Differences in Transcriptional Profiles Between AM and MDM

**Figure 2:**
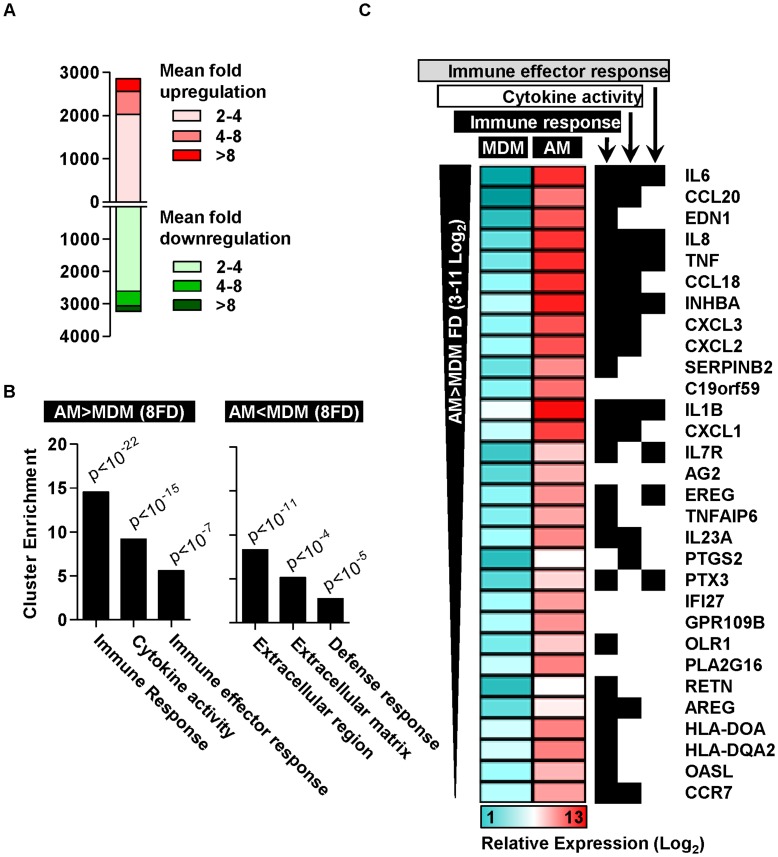
Differences in transcriptional profiles of AM and MDM. (A) Comparison of transcriptional profiles of AM and MDM shows extensive gene expression differences. (B) Functional annotation clustering of significant gene expression differences (>8-fold and p<0.05, t-test) shows that genes upregulated in AM are highly enriched for immune responses and cytokine activity, whereas downregulated genes show less significant enrichment for defense responses and extracellular processes (Modified Fisher’s Exact Test). (C) The three most significantly enriched gene ontology associations are shown for the top 30 most highly upregulated genes in AM.

Next we sought to make a comprehensive comparison of AM and MDM using genome-wide expression data. This analysis showed extensive gene expression differences (Figure 2A). In order to explore these differences at a systems level, we conducted functional annotation clustering analysis by gene ontology associations for significant gene expression differences. In this analysis, we focussed on genes with the greatest differential expression by including all those showing greater than 8-fold difference. Genes that were more highly expressed in AM, were significantly enriched for immune, inflammatory and defense responses and cytokine activity (Table 1 and Figure 2B). These findings contrasted with genes that were more highly expressed in MDM which showed much less significant ontological enrichment for soluble mediator and defense response processes (Figure 2B). Further analysis of the most highly upregulated genes in AM compared with MDM confirmed marked enrichment for immune responses and cytokines (Figure 2C and Figure S1A). Investigation of the transcriptional regulation underlying these gene expression differences by transcription factor binding site analysis of genes which were more highly expressed by more than eight-fold in AM, showed highly significant enrichment for NF-κB family members (Table 2), which are key regulators of pro-inflammatory gene expression. These data clearly show that freshly isolated AM exhibit a wide ranging pro-inflammatory transcriptional profile.

**Table 1:** Ontological associations of gene expression differences between AM and MDM.

Term	% of gene list	p value*	Fold Enrichment
GO:0006955∼immune response	19.2	7.92E^−22^	4.6
GO:0009611∼response to wounding	15.5	3.86E^−18^	4.8
GO:0006954∼inflammatory response	12.0	3.56E^−17^	6.1
GO:0006952∼defense response	15.8	1.86E^−16^	4.2
GO:0005125∼cytokine activity	8.9	2.05E^−15^	7.9
GO:0005615∼extracellular space	14.8	2.15E^−14^	3.9
GO:0044421∼extracellular region part	15.8	2.74E^−11^	3.0
GO:0005576∼extracellular region	21.3	1.76E^−07^	1.9

* Modified Fisher’s Exact Test.

Functional Annotation Clustering analysis by gene ontology classification of significant gene expression differences identified by transcriptional profiling of unstimulated AM (4 donors) and MDM (8 donors).

**Table 2:** Transcription factor enrichment of highly upregulated genes in AM.

Transcription factor	Z-score†
RELA	30.52
NF-kappaB	26.15
REL	25.55
HLF	20.72
NFKB1	19.54
MEF2A	14.23
NFIL3	13.08
SRY	12.81
ESR1	12.15
STAT1	12.08
CREB1	12.04
ELF5	11.5
SRF	11.4
ELK1	10.73

† z-scores of >10 are considered to indicate highly significant over-representation of transcription factor binding sites within the analysed gene list.

### Pro-inflammatory Phenotype of Freshly Isolated AM

**Figure 3:**
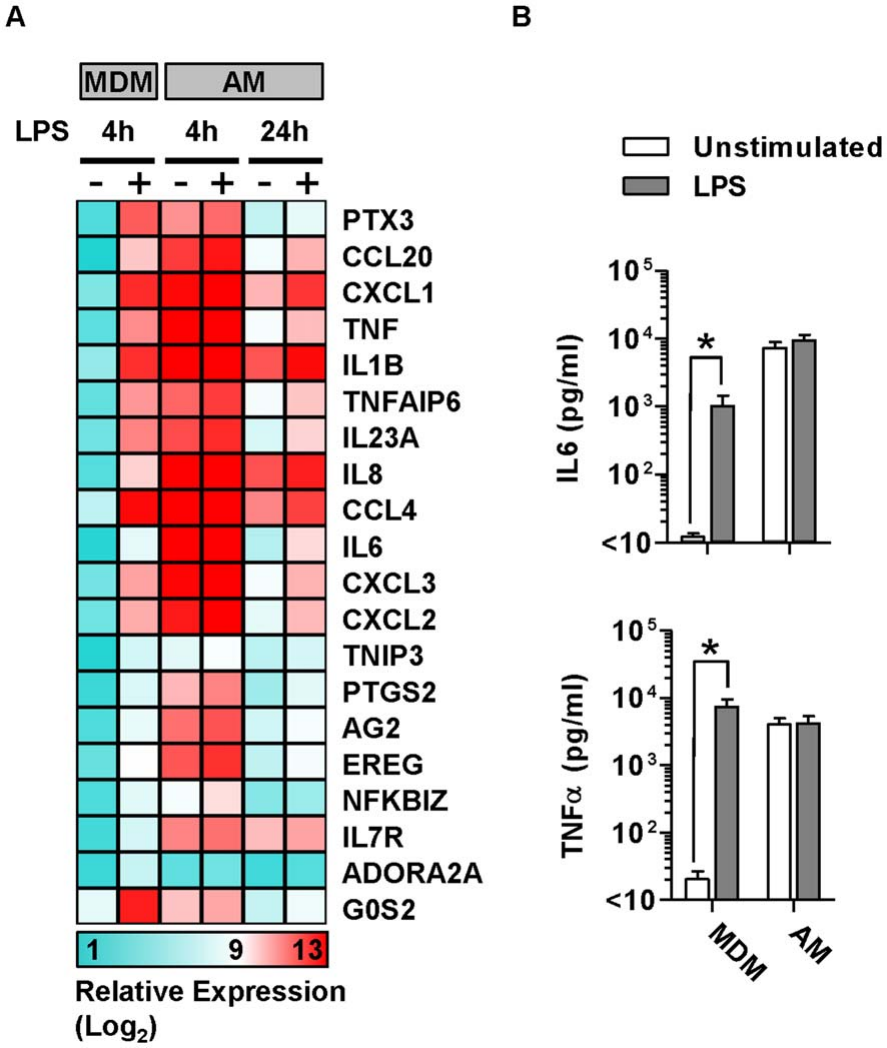
Inflammatory responses by freshly isolated AM. (A) Mean relative gene expression levels are presented in MDM and AM before and after 4–24 hour LPS stimulation, for the top 20 most upregulated genes following four hour LPS stimulation of MDM. (B) AM show extremely high baseline expression of pro-inflammatory genes which is not further upregulated following four hour LPS stimulation, however, after 24 hours in culture, basal inflammatory gene levels are reduced in AM, and LPS induced upregulation becomes evident. High basal pro-inflammatory cytokine levels which are not increased by LPS stimulation are also present in AM culture supernatants. Bars represent mean ± SEM for at least 4 separate experiments (*denotes p<0.05, t-test).

Macrophages play a key role in innate immune inflammatory responses, which are predominantly reflected by changes in gene expression. Therefore we tested transcriptional responses to innate immune stimulation of AM and compared these to MDM. Four hour stimulation of MDM with LPS induced upregulated expression of a well validated set of cytokines and chemokines, but in freshly isolated AM all of these genes showed high level expression at baseline and no further upregulation after LPS stimulation (Figure 3A). In order to establish if the gene expression data were reflected at the protein level, we measured the levels of two prototypic inflammatory cytokines, tumour necrosis factor (TNF)α and interleukin (IL)6 in cell culture supernatants of freshly isolated AM and MDM before and after stimulation with LPS (Figure 3B). In MDM cultures, LPS induced significant increases in both TNFα and IL6. In keeping with the gene expression data, both of these cytokines were present at high concentrations in freshly isolated AM cultures and did not increase further in response to LPS stimulation, suggesting that the pro-inflammatory capacity of AM was already maximally activated.

### Baseline Pro-inflammatory Responses by AM Diminish with Time in Culture

**Figure 4:**
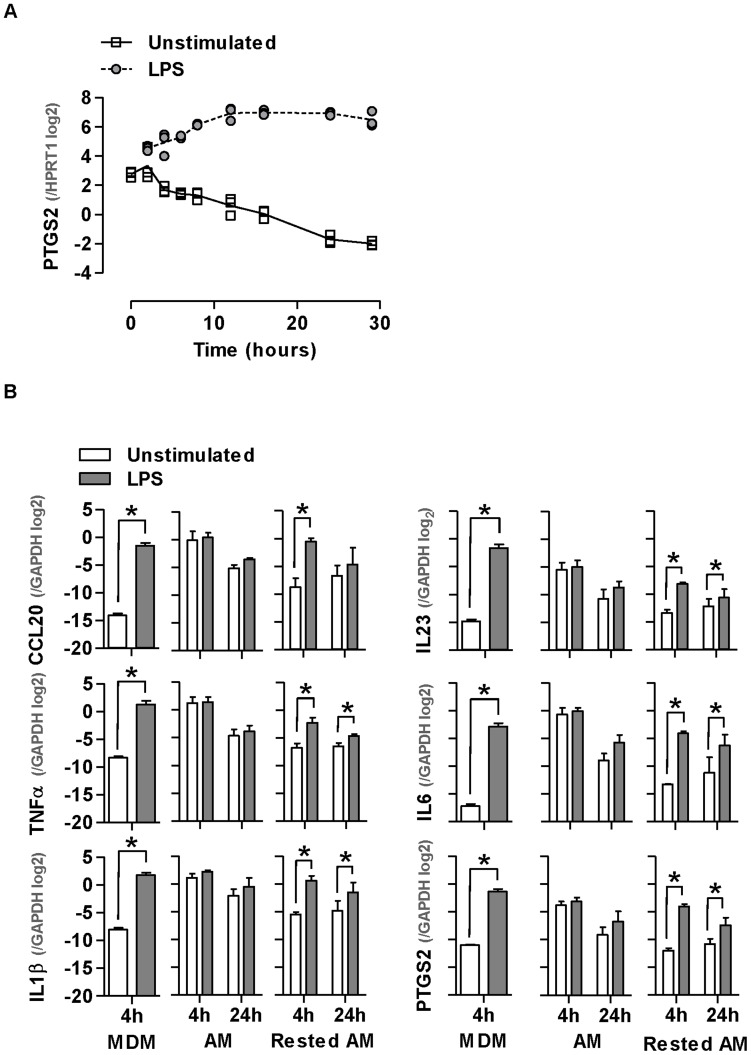
Reduction of AM inflammatory gene expression with time in culture. (A) AM show a significant reduction in PTGS2 expression over time (p<0.0001, ANOVA). Data points represent technical replicates of measurements from one patient with DIP. (B) Selected gene expression changes identified by microarrays are validated by qPCR. Basal pro-inflammatory gene expression is reduced in AM which have been rested for 48 hours and upregulated following LPS stimulation. Bars represent mean ±SEM for at least 4 separate experiments (*denotes p<0.05, t-test).

In our gene array analysis, we noted that the expression of inflammatory genes was lower in unstimulated AM cultured for 24 hours, when compared to cells cultured for 4 hours (Figure 3A). Interestingly as basal expression levels for these genes diminished at 24 hours, upregulation following LPS stimulation also became evident. In order to assess the effect of time in culture further, we used cells obtained from a patient with desquamative interstitial pneumonia, characteristically associated with very high AM yields from BALF [15], providing an opportunity to perform a detailed time course expression analysis of a representative inflammatory gene-PTGS2, from 0–28 hours. This analysis showed that basal PTGS2 expression levels progressively reduced over time in culture, such that changes to gene expression levels following LPS stimulation became more apparent (Figure 4A).

We extended these studies to test the expression levels of a panel of six inflammatory genes in AM from healthy participants, which were either used immediately or rested in culture for 48 hours before LPS stimulation for 4–24 hours (Figure 4B). In all cases basal pro-inflammatory gene expression levels in rested AM were significantly lower than in freshly isolated AM, and comparable to levels in unstimulated MDM. Furthermore, upregulated expression of these pro-inflammatory genes following LPS stimulation was only evident in rested AM, and the same observations were evident using an alternative stimulus, the RNA analogue poly I:C, commonly used to probe anti-viral innate immune responses (Figure S2). Collectively, these results show that freshly isolated AM exhibit a highly pro-inflammatory profile at baseline, with consequent lack of further capacity to respond to innate immune stimulation, both of which are partially reversed with time in culture.

### Gene Expression Profiles of Rested AM Approximate More Closely to MDM, but Show Persistent Upregulation of Components of the MHC Class II Antigen Presentation Pathway

**Figure 5:**
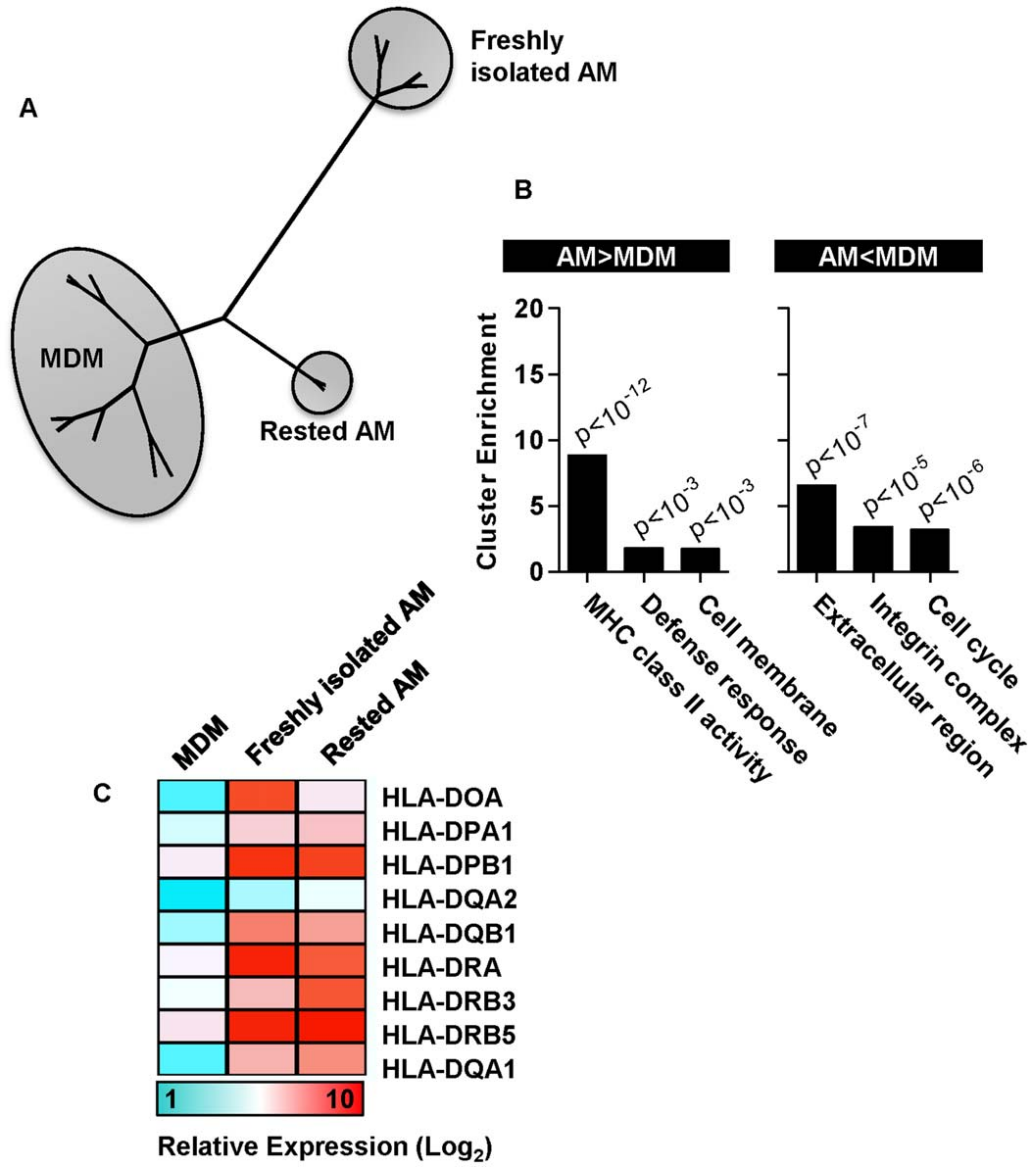
Genome-wide comparison of MDM with freshly isolated and rested AM. (A) Hierarchical clustering of whole genome expression profiles showed greatest difference between MDM and freshly isolated AM, and closer clustering of rested AM to MDM. (B) Significant gene expression differences between rested AM and MDM assessed by gene ontology functional cluster analysis, showed greatest enrichment for upregulated expression in AM of genes associated with MHC class II antigen presentation pathways. (C) Mean relative gene expression levels are presented in MDM and AM for genes included in this cluster.

In view of the reduction of pro-inflammatory gene expression in rested AM to comparable levels seen in MDM, we assessed whether rested AM approximated more closely to MDM at genome-wide level also. Hierarchical clustering of whole genome expression data, showed that rested AM grouped more closely to MDM than freshly isolated AM but still formed a distinct cluster (Figure 5A). We therefore assessed the residual gene expression differences between rested AM and MDM. Gene ontology functional cluster analysis of these differences showed most significant enrichment for increased expression of genes associated with MHC class II activity in AM (Figure 5B–C).

## Discussion

AM are widely considered to be important cellular constituents of the lower respiratory tract and by analogy to macrophage biology in general, are thought to play a key role in innate immune recognition of microbial pathogens or other danger signals, generation of inflammatory responses, control of intracellular pathogens and homeostatic regulation of immune responses. They also serve as a bridge between innate and adaptive immunity through antigen presentation and provision of co-stimulatory signals. MDM are commonly used to model AM, but few studies have directly compared human AM with MDM, hence we have very little genuine insight into the specific phenotype and function of AM. In the present study we used genome-wide transcriptional profiling to compare AM with MDM and focussed specifically on transcriptional responses to innate immune stimulation with LPS. We obtained AM from patients undergoing bronchoscopy for reported haemoptysis, but found to have normal radiology and no other evidence of respiratory tract pathology after follow up. Nonetheless it is possible that these selection criteria may have affected our findings. In addition, cigarette smoking has been reported to affect gene expression profiles in AM from different subjects [16], but our focus was comparison of AM with MDM, therefore we included both smokers and non-smokers to avoid any systematic confounding of the data as a result of smoking status.

We demonstrate that freshly isolated AM bear the major transcriptional features of MDM, but AM showed greatly increased expression of genes strongly associated with pro-inflammatory responses. Although these data conflict with a widely held view that AM have a predominant anti-inflammatory or regulatory phenotype [7], [12], similar upregulation of immune and defense response genes were noted in a previous microarray study [17], but without systematic analysis of the significance of reported gene expression differences or consideration of the possible implications. The possibility that AM exhibit a steady state pro-inflammatory phenotype, is further supported by the finding that spontaneous inflammatory cytokine (IL6 and IL8) production by human lung tissue explants cultured *ex vivo*, was reduced by depletion of AM with clodronate [4], and by additional studies that show spontaneous secretion of pro-inflammatory cytokines by AM from healthy volunteers [18]–[20], albeit not a consistent finding in the literature. Clearly, the isolation procedure may have resulted in cellular activation, as illustrated by induction of gene expression changes in AM after enrichment by adherence [21]. Alternatively, the intra-alveolar microenvironment is reported to be biased towards an anti-inflammatory phenotype in order to limit damaging inflammatory responses [12], therefore removal of AM from local inhibitory factors *in vivo*, such as prostaglandin E2 (PGE2), TGFβ or surfactant, which have all been shown to attenuate AM inflammatory mediator production, may account for our *ex vivo* observations [22]–[24]. In view of our findings, and the established role of the pro-inflammatory growth factor GM-CSF in AM biology, we hypothesize that AM have the capacity to adopt a striking pro-inflammatory profile, which may be highly regulated by their *in vivo* tissue microenvironment. In order to test this hypothesis further studies on tissue samples from the lung are necessary to establish whether AM show any features of a pro-inflammatory phenotype in situ.

Importantly, freshly isolated AM did not further upregulate pro-inflammatory gene expression following innate immune stimulation. However, pro-inflammatory mediator expression progressively declined with time in culture, such that by 24 hours, LPS and poly I:C stimulated upregulation of these genes was discernible. These findings are consistent with previous reports of inflammatory cytokine induction by AM after LPS stimulation for 18–24 hours [18], [20], [21]. We extended these observations with further novel data showing that basal pro-inflammatory gene expression was reduced in AM which were rested in culture for 48 hours, and similar to levels detected in MDM, with concomitant restoration of transcriptional responsiveness to innate immune stimulation. Therefore, by contrast to the common use of AM immediately following isolation and enrichment by adherence (Table S3), such investigations may be better conducted on rested cells. The highly activated phenotype of freshly isolated AM may still significantly confound responses to inflammatory stimuli and merits careful consideration during experimental design and data interpretation in future studies.

Transcriptional profiles from rested AM were clearly more similar to MDM than freshly isolated AM, but still represented by a distinct cluster. Interestingly the most highly enriched group of genes that were persistently upregulated in AM compared to MDM, were MHC II antigen molecules. This finding is consistent with previous reports of high MHC II expression in AM that may also be attributed to the role of GM-CSF in AM biology [9], [25]. This assessment at genome-wide level suggests that antigen presentation capacity may represent a key functional difference between AM and MDM, and merits further investigation.

## Materials and Methods

### Ethics Statement

The study was approved by the joint University College London/University College Hospitals National Health Service Trust Human Research Ethics Committee, and written informed consent was obtained from all participants.

### Cell isolation and Culture

Blood samples were obtained from healthy volunteers for isolation of PBMC and production of MDM by differentiation with M-CSF as previously described [26]. AM were obtained from patients undergoing bronchoscopy for investigation of possible haemoptysis, in the context of normal thoracic computed tomography (CT) scanning and from one patient with desquamative interstitial pneumonia (Table S1). CD14 positive monocytes were isolated from PBMC by magnetic cell sorting (Miltenyi Biotec) according to the manufacturer’s instructions. Monocytes were differentiated into dendritic cells (DC) as previously described [27]. AM were isolated by BAL. Three 60 ml aliquots of warmed normal saline were instilled via a wedged fibreoptic bronchoscope, aspirated BAL fluid was immediately cooled to 4°C and filtered through a cell strainer to remove particulate debris before centrifugation. AM enrichment and culture were performed using a standard protocol [28]. Briefly, BAL cells were resuspended in AM medium- RPMI 1640 with L-glutamine containing 5% heat inactivated pooled human type AB serum, 100 IU/ml penicillin and 100 µg/ml streptomycin (Invitrogen), and seeded at 5×10^5^ cells per well in 12 well plates (TPP), removing non-adherent cells after one hour. Ultrapure LPS (100 ng/ml) from *Escherichia coli O111:B4* and poly IC (10 µg/ml), both from Invivogen, were used to stimulate inflammatory responses. All cell lines were obtained from the American Type Culture Collection (ATCC). THP1 and SUPT1 cell lines were cultured as suspension cells in RPMI and HeLa cells as adherent cells in DMEM with 10% foetal calf serum and antibiotics (as above). THP1 cells were differentiated by addition of 5 ng/mL phorbol myristate acetate (Sigma Aldrich) for 72 hours [29].

### Transcriptonal Profiling by cDNA Microarray

Total RNA was purified from cell lysates collected in RLT buffer (Qiagen) using the RNEasy Mini kit (Qiagen). Human universal standard reference RNA representing an equal mixture of ten human cell lines was obtained from Stratagene. Samples were processed for Agilent microarrays and data were normalised as previously described [30]. T tests were used to identify significant gene expression differences (p<0.05) between samples. Relative differences in transcriptional profiles were compared by hierarchical clustering by Euclidean distance, using MultiExperiment Viewer v4.6.0 and dendograms were created using TreeView v1.6.6. DAVID functional annotation clustering was used to annotate gene lists of interest by gene ontology associations and transcriptional regulation of specific gene expression profiles was assessed by single transcription factor binding site enrichment analysis as previously described [27]. Microarray data are available in the ArrayExpress database under accession number E-TABM-1206.

### Quantitative PCR

cDNA was synthesised using the qScript cDNA Supermix kit (Quanta BioSciences) and quantitative (q)PCR of selected genes was performed using the following inventoried TaqMan assays (Applied Biosystems); HPRT1 (Hs99999909_m1), CCL20 (Hs01011368_m1), IL23 (Hs00372324_m1), IL1β (Hs01555410_m1), IL6 (Hs00985639_m1) and TNFα (Hs00174128_m1). PTGS2 expression was quantified using: forward primer: CGGTCCTGGCGCTCAG, reverse primer: CCGGGTACAATCGCACTTATACTG, and probe: CCATACAGCAAATCCTT. Expression levels of target genes were normalised to that of HPRT1 (Hs99999909_m1) or glyceraldehyde-3-phosphate dehydrogenase (GAPDH) as previously described [26].

### Cytokine Measurements

MDM and AM culture supernatant cytokine concentrations were quantified by ELISA (eBioscience) according to the manufacturer’s instructions.

## Supporting Information

Figure S1 **Gene ontology associations of expression differences between AM and MDM.** The top three most significantly enriched gene ontology associations are presented for the 50 most highly (A) upregulated and (B) downregulated genes in AM compared with MDM.
(TIF)

Figure S2 **Poly I:C induced pro-inflammatory gene expression in rested AM.** Freshly isolated alveolar macrophages (AM) show no pro-inflammatory transcriptional response to stimulation with poly IC (10 µg/ml), but basal levels of pro-inflammatory gene expression are reduced in AM which have been rested for 48 hours and these show significant upregulation following 4 hour stimulation with poly IC (10 µg/ml). Bars represent mean ±SEM for 3 separate experiments (*denotes p<0.05, t-test).
(TIF)

Table S1 **Clinical information for AM donors.** Patient details for samples used for freshly isolated AM arrays^*^ and ELISAs^‡^, time course study of PTGS2 expression^†^ and 48 hour rested AM^§^. DIP =  desquamative interstitial pneumonia.
(DOC)

Table S2 **Supporting literature for macrophage transcriptional signature.** In genome-wide expression data, genes that showed significantly higher expression (>8 fold) in monocyte derived macrophages compared to standard reference RNA were subjected to PubMed searches to identify those that are known to be expressed by macrophages, and thereby generate a transcriptional signature for macrophages. The first author, publication year, journal, direct object identifier (DOI) and PubMed identifier (PMID) is provided for representative citations associated with each of these genes.
(DOC)

Table S3 **Summary of AM enrichment and culture methods.** Recently published studies involving alveolar macrophages were identified from PubMed using the following search criteria: “(((((macrophages) AND human) AND bronchoalveolar lavage) NOT mouse) NOT murine) NOT mice” with Limits: Humans, Journal Article, English, published in the last 5 years. The method of AM enrichment and the time in culture before AM were assessed is indicated for each study, reflecting the common contemporary practice of enrichment by plastic adherence before use of freshly isolated AM. The first author, publication year, journal, direct object identifier (DOI) and PubMed identifier (PMID) is provided for each study.
(DOC)
