# Supplementary figures and images for: Adherent Human Alveolar Macrophages Exhibit a Transient Pro-Inflammatory Profile That Confounds Responses to Innate Immune Stimulation
Source: PLoS One. 2012 Jun 29;7(6):e40348. doi: 10.1371/journal.pone.0040348 (PMC3386998; doi:10.1371/journal.pone.0040348)

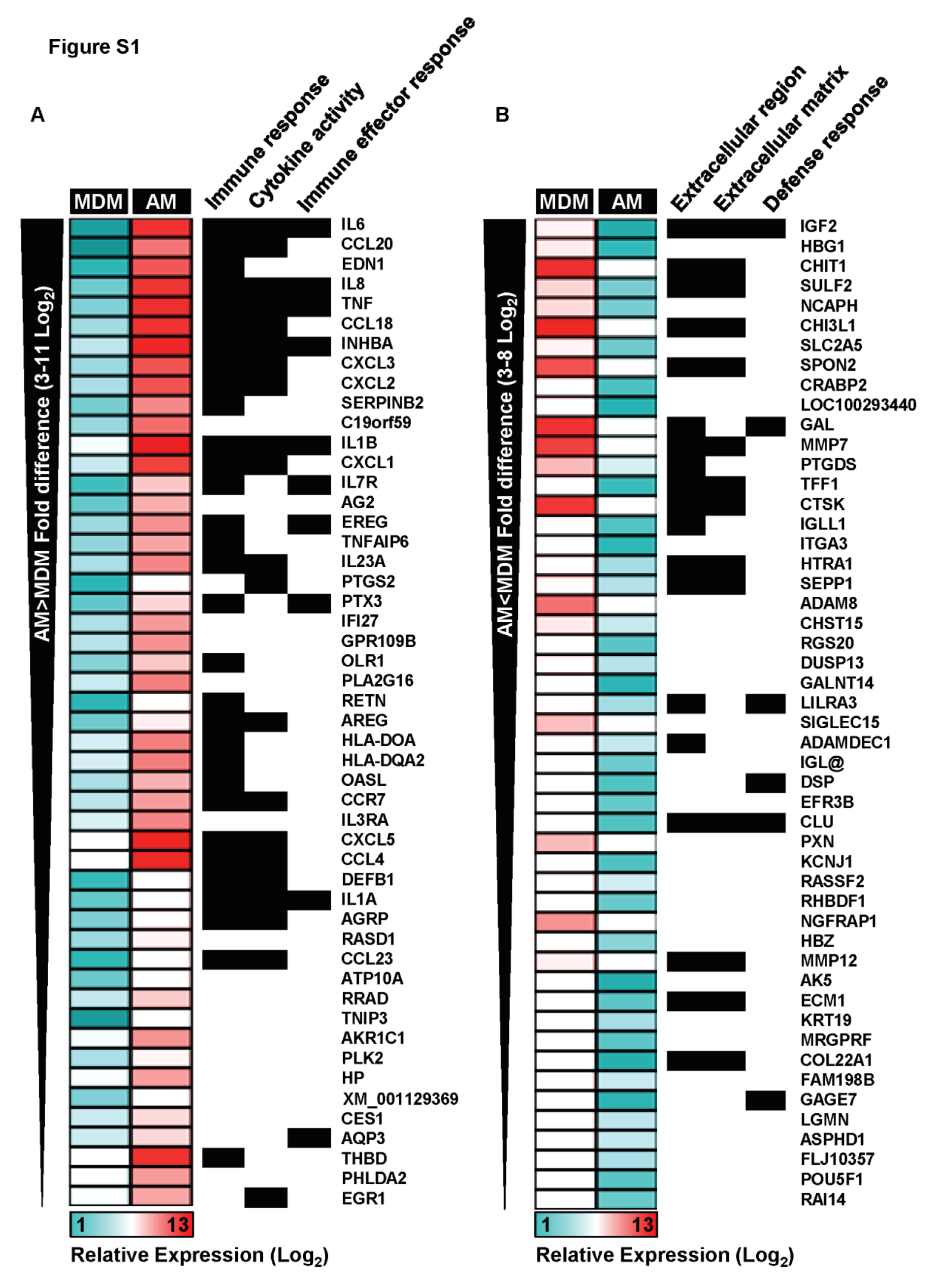

Supplement: Figure S1 — Gene ontology associations of expression differences between AM and MDM. The top three most significantly enriched gene ontology associations are presented for the 50 most highly (A) upregulated and (B) downregulated genes in AM compared with MDM. (TIF) [file pone.0040348.s001.tif]

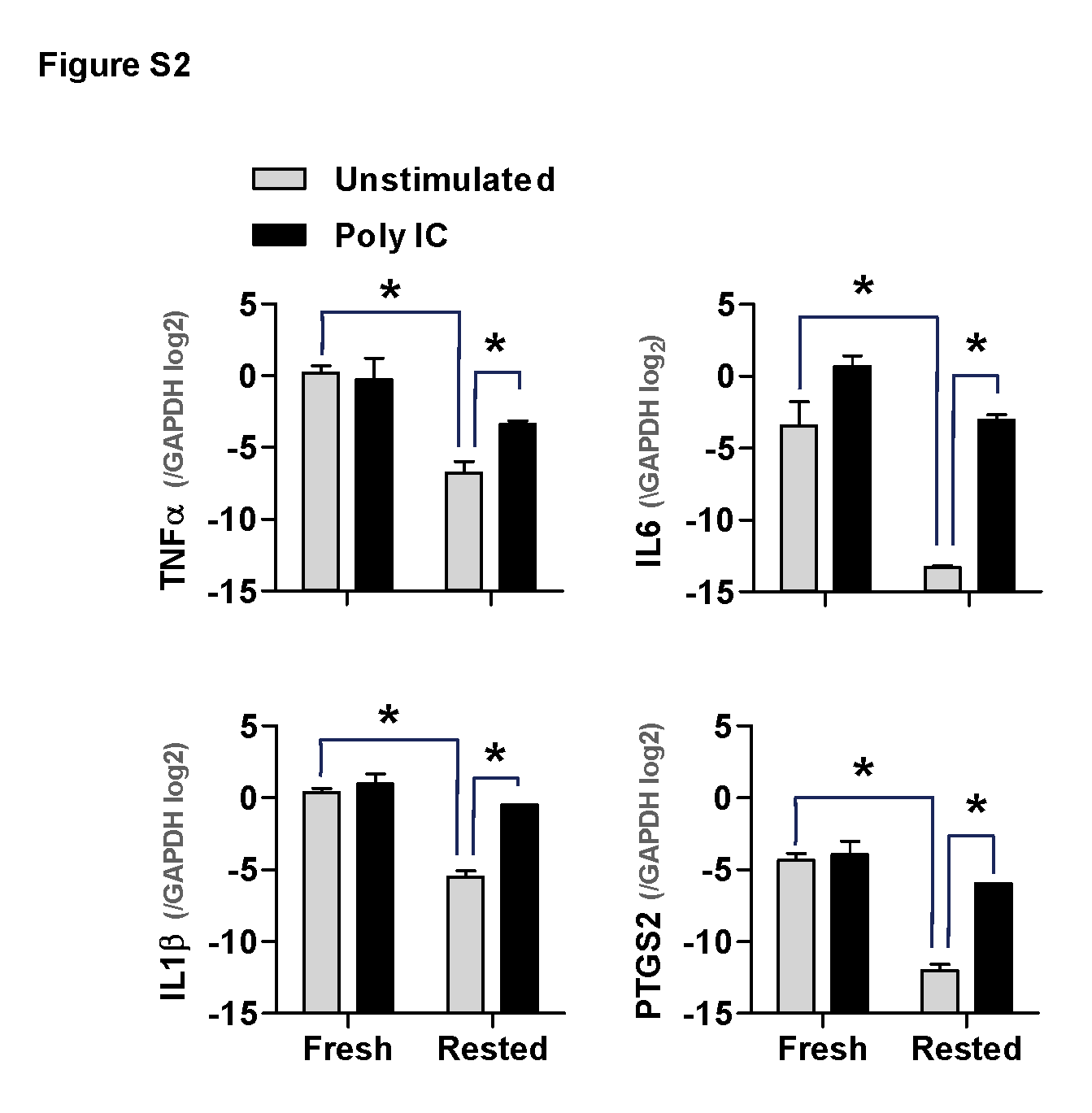

Supplement: Figure S2 — Poly I:C induced pro-inflammatory gene expression in rested AM. Freshly isolated alveolar macrophages (AM) show no pro-inflammatory transcriptional response to stimulation with poly IC (10 µg/ml), but basal levels of pro-inflammatory gene expression are reduced in AM which have been rested for 48 hours and these show significant upregulation following 4 hour stimulation with poly IC (10 µg/ml). Bars represent mean ±SEM for 3 separate experiments (*denotes p<0.05, t-test). (TIF) [file pone.0040348.s002.tif]
