# Supplementary material for: Adherent Human Alveolar Macrophages Exhibit a Transient Pro-Inflammatory Profile That Confounds Responses to Innate Immune Stimulation
Source: PLoS One. 2012 Jun 29;7(6):e40348. doi: 10.1371/journal.pone.0040348 (PMC3386998; doi:10.1371/journal.pone.0040348)
Supplement: Table S1 — Clinical information for AM donors. Patient details for samples used for freshly isolated AM arrays* and ELISAs‡, time course study of PTGS2 expression† and 48 hour rested AM§. DIP = desquamative interstitial pneumonia. (DOC) [file pone.0040348.s003.doc]

**Table S1**

| **Sample ID** | **Age** | **Gender** | **Ethnicity** | **FEV1 (%)** | **FVC (%)** | **Smoking** | **Pack years** | **Medication** | **Diagnosis** |
| --- | --- | --- | --- | --- | --- | --- | --- | --- | --- |
| B271**^†^** | 44 | F | White | 101 | 109 | Current | 33 | Beclomethasone inhaler | DIP |
| B302**^‡^** | 51 | M | White | N/A | N/A | Ex | 20 | None | None |
| B306**^*^** | 46 | M | African | N/A | N/A | Current | 30 | None | None |
| B312**^*‡^** | 44 | F | White | N/A | N/A | Ex | 10 | None | None |
| B313**^‡^** | 35 | M | White | N/A | N/A | Never | N/A | None | None |
| B315**^‡^** | 66 | F | White | N/A | N/A | Current | 25 | None | None |
| AMI 2**^*‡^** | 77 | M | White | N/A | N/A | Current | 50 | None | None |
| AMI 3**^*^** | 48 | F | White | 97 | 113 | Current | 20 | Beclomethasone inhaler | None |
| AMI 6**^§^** | 53 | F | White | 95 | 98 | Never | N/A | None | None |
| AMI 7**^§^** | 23 | F | Asian Indian | 75 | 87 | Never | N/A | None | None |
| AMI 8**^§^** | 44 | M | White | N/A | N/A | Current | 40 | None | None |
