# Supplementary material for: Adherent Human Alveolar Macrophages Exhibit a Transient Pro-Inflammatory Profile That Confounds Responses to Innate Immune Stimulation
Source: PLoS One. 2012 Jun 29;7(6):e40348. doi: 10.1371/journal.pone.0040348 (PMC3386998; doi:10.1371/journal.pone.0040348)
Supplement: Table S2 — Supporting literature for macrophage transcriptional signature. In genome-wide expression data, genes that showed significantly higher expression (>8 fold) in monocyte derived macrophages compared to standard reference RNA were subjected to PubMed searches to identify those that are known to be expressed by macrophages, and thereby generate a transcriptional signature for macrophages. The first author, publication year, journal, direct object identifier (DOI) and PubMed identifier (PMID) is provided for representative citations associated with each of these genes. (DOC) [file pone.0040348.s004.doc]

**Table S**2

| **Gene Name** | **Author** | **Year** | **Journal** | **DOI** | **PMID** |
| --- | --- | --- | --- | --- | --- |
| ADAMDEC1 | Fritsche | 2003 | Immunology | N/A | 14632642 |
| CCR5 | Kaufmann | 2001 | J Leukocyte Biol | N/A | 11272275 |
| CD14 | Greaves | 2002 | Int J Haematol | N/A | 12138897 |
| CD36 | Huh | 1996 | Blood |  | 8634453 |
| CD68 | Greaves | 2002 | Int J Haematol | N/A | 12138897 |
| CD163 | Fabriek | 2009 | Blood | 10.1182/blood-2008-07-167064 | 18849484 |
| CSF1R | Heusohn | 1994 | Cell Growth Differ | N/A | 7848913 |
| CTSD | Diment | 1988 | J Biol Chem | N/A | 3360812 |
| EGR2 | Pospisil | 2011 | EMBO J | 10.1038/emboj.2011.317 | 21897363 |
| MARCO | Kraal | 2000 | Microbes Infect | S1286-4579(00)00296-3 | 10758408 |
| MNDA | Briggs | 1994 | J Cell Biochem | 10.1002/jcb.240560417 | 7890814 |
| MSR1 | Greaves | 2002 | Int J Haematol | N/A | 12138897 |
