# Supplementary material for: Adherent Human Alveolar Macrophages Exhibit a Transient Pro-Inflammatory Profile That Confounds Responses to Innate Immune Stimulation
Source: PLoS One. 2012 Jun 29;7(6):e40348. doi: 10.1371/journal.pone.0040348 (PMC3386998; doi:10.1371/journal.pone.0040348)
Supplement: Table S3 — Summary of AM enrichment and culture methods. Recently published studies involving alveolar macrophages were identified from PubMed using the following search criteria: “(((((macrophages) AND human) AND bronchoalveolar lavage) NOT mouse) NOT murine) NOT mice” with Limits: Humans, Journal Article, English, published in the last 5 years. The method of AM enrichment and the time in culture before AM were assessed is indicated for each study, reflecting the common contemporary practice of enrichment by plastic adherence before use of freshly isolated AM. The first author, publication year, journal, direct object identifier (DOI) and PubMed identifier (PMID) is provided for each study. (DOC) [file pone.0040348.s005.doc]

**Table S3**

| **Author** | **Year** | **Journal** | **DOI** | **PMID** | **AM enrichment method** | **Time** |
| --- | --- | --- | --- | --- | --- | --- |
| Laza-Stanca | 2011 | PLoS Pathog | 10.1371/journal.ppat.1002114 | 21779162 | None | < 24 hours |
| Liang | 2011 | J Allergy Clin Immunol | 10.1016/j.jaci.2011.04.006 | 21570715 | Plastic adherence | 24-48 hours |
| Burnham | 2011 | Alcohol Clin Exp Res | 10.1111/j.1530-0277.2010.01344.x | 21121937 | None | < 24 hours |
| Wu | 2010 | Can J Microbiol | 0.1139/W10-002 | 20453909 | Plastic adherence | 24-48 hours |
| Ferhani | 2010 | [Am J Respir Crit Care Med.](../American%20journal%20of%20respiratory%20and%20critical%20care%20medicine.) | 10.1164/rccm.200903-0340OC | 20133931 | Plastic adherence | 24-48 hours |
| Juarez | 2010 | Respir Res | 10.1186/1465-9921-11-2 | 20051129 | None | < 24 hours |
| Chen | 2010 | Am J Respir Crit Care Med | 10.1164/rccm.200905-0696OC | 19910611 | None | < 24 hours |
| Hoogerwerf | 2010 | Am J Respir Cell Mol Biol | 10.1165/rcmb.2008-0362OC | 19448156 | CD71 MACS beads isolation | < 24 hours |
| Armstrong | 2009 | Clin Exp Immunol | 10.1111/j.1365-2249.2009.03906.x | 19292764 | Plastic adherence | < 24 hours |
| Message | 2008 | PNAS | 10.1073/pnas.0804181105 | 18768794 | None | < 24 hours |
| Létuvé | 2008 | J Immunol | N/A | 18802121 | Plastic adherence | 24-48 hours |
| Goleva | 2008 | J Allergy Clin Immunol | 1016/j.jaci.2008.07.007 | 18774390 | None | < 24 hours |
| Kazeros | 2008 | Am J Respir Cell Mol Biol | 10.1165/rcmb.2007-0306OC | 18587056 | Plastic adherence | 24-48 hours |
| Nicol | 2008 | J Virol | 10.1128/JVI.00362-08 | 18524817 | Plastic adherence | 24-48 hours |
| Hoogerwerf | 2008 | Am J Respir Crit Care Med | 10.1164/rccm.200708-1261OC | 18403723 | CD71 MACS beads isolation | < 24 hours |
| Faith | 2008 | Allergy | 10.1111/j.1398-9995.2007.01531.x | 18186807 | Plastic adherence | < 24 hours |
| Saint-Georges | 2008 | Toxicology | 10.1016/j.tox.2007.11.016 | 18178302 | Plastic adherence | < 24 hours |
| [Tudhope](http://jpet.aspetjournals.org/search?author1=Susan+J.+Tudhope&sortspec=date&submit=Submit) | 2008 | J Pharmacol Exp Ther | 10.1124/jpet.107.127670 | 17921189 | Plastic adherence | 24-48 hours |
| Mwandumba | 2007 | Microbes Infect | 10.1016/j.micinf.2007.04.013 | 17644388 | None | < 24 hours |
| Wygrecka | 2007 | Thorax | 10.1136/thx.2006.069658 | 17483138 | Plastic adherence | < 24 hours |
| Hoshino | 2007 | J Infect Dis | 10.1086/513438 | 17396999 | Plastic adherence | < 24 hours |
| Rotoli | 2007 | Am J Respir Cell Mol Biol | 10.1165/rcmb.2006-0262OC | 17363779 | Plastic adherence | < 24 hours |
| Sable | 2007 | Eur Respir J | 10.1183/09031936.00111205 | 17079254 | Sheep RBC & neuraminidase | < 24 hours |
| Kauth | 2007 | Int Arch Allergy Immunol | 10.1159/000096381 | 17057409 | Plastic adherence | < 24 hours |
| Li | 2007 | J Leukoc Biol | N/A | 17046970 | Plastic adherence | < 24 hours |
